# Bardet-Biedl syndrome caused by compound heterozygosity in BBS12 gene: a case report of one family with three affected members

**DOI:** 10.3389/fped.2023.1226595

**Published:** 2023-07-04

**Authors:** Ana Simičić Majce, Darija Tudor, Marko Simunovic, Marko Todorovic, Mladenka Parlov, Bernarda Lozic, Mirna Saraga-Babić, Marijan Saraga, Adela Arapović

**Affiliations:** ^1^ Paediatric Diseases Department, University Hospital of Split, Spinciceva 1, Split, Croatia; ^2^ University of Split School of Medicine, Soltanska 2, Split, Croatia; ^3^ Physical Medicine and Rehabilitation with Rheumatology Division, University Hospital of Split, Spinciceva 1, Split, Croatia

**Keywords:** Bardet-Biedl syndrome, mutation, ciliopathies, kidney disease, family

## Abstract

**Introduction:**

Bardet-Biedl syndrome (BBS) is a rare genetic syndrome caused by a mutation in one of 26 different genes responsible for normal structure and/or function of primary cilia. The syndrome is characterized by multiorgan involvement with gradual onset of occurrence of clinical signs and symptoms resulting in great phenotypic variability and what is more important, often difficulties with establishing the timely diagnosis.

**Case report:**

We report a case of a one family with three members with BBS caused by a very rare mutation, a compound heterozygosity in *BB12* gene. Even though all three patients have the same type of mutation, they express a significant diversity in clinical expression as well as renal impairment.

**Conclusion:**

This is a case report of a rare clinical syndrome caused by a very rare genetic mutation and it emphasizes the importance of genetic analysis in the timely diagnosis of oligosymptomatic patients with BBS, in order to possibly prevent long-term complications.

## Introduction

Bardet-Biedl syndrome (BBS) is a rare autosomal recessive ciliopathy that can be caused by a mutation in at least 26 different genes. Clinical presentation varies from signs present at birth such as polydactyly, syndactyly, and brachydactyly to the development of obesity, metabolic syndrome, retinopathy, as well as renal, cardiac, gastrointestinal, neural, and genitourinary abnormalities that develop over time (1). Although at least four major criteria (or three major and two minor) must be met for a clinical diagnosis, the gradual development of individual clinical signs during childhood combined with variations in the genotype-phenotype correlation indicate an increasing number of “genetically positive” BBS patients who do not meet the mentioned criteria, which is also confirmed by recent meta-analyses (2, 3). Major features include cone-rod dystrophy, central (truncal) obesity, postaxial polydactyly, cognitive impairment, hypogonadism, genitourinary malformations and kidney disease while minor features include neurodevelopmental abnormalities, dysmorphic craniofacial features, anosmia, orodental abnormalities, cardiovascular, gastrointestinal and endocrine abnormalities. The genetic background of BBS is an autosomal recessive inherited mutation in one of the 26 detected genes associated with this disorder that results in biallelic loss of function (1, 2). We report a rare case of a family with three members with BBS. Our patients were a male newborn, his brother and his sister. Genetic analysis revealed that they are compound heterozygotes for the *BBS12* gene. It is interesting to point out that mutations in *BBS12* gene are responsible for approximately 8% of all cases of Bardet Biedl syndrome reported in the literature (4). Furthermore, this case report represents a very rare genetic substrate underlying this syndrome – compound heterozygosity, which implies two different mutations in each allele of *BBS12* gene with distinctive clinical expression. Even though there is a certain level of genotype to phenotype correlation in BBS, our patients do not have a complete overlap of the clinical presentation (3). The question arises whether the detection of a certain genotype can also be a predictor of the development of a particular phenotype over time and to what extent. The above is not negligible considering that certain components of this clinical syndrome determine the quality of life and bear significant morbidity and mortality of patients with BBS (5).

## Results

**Figure 1:**
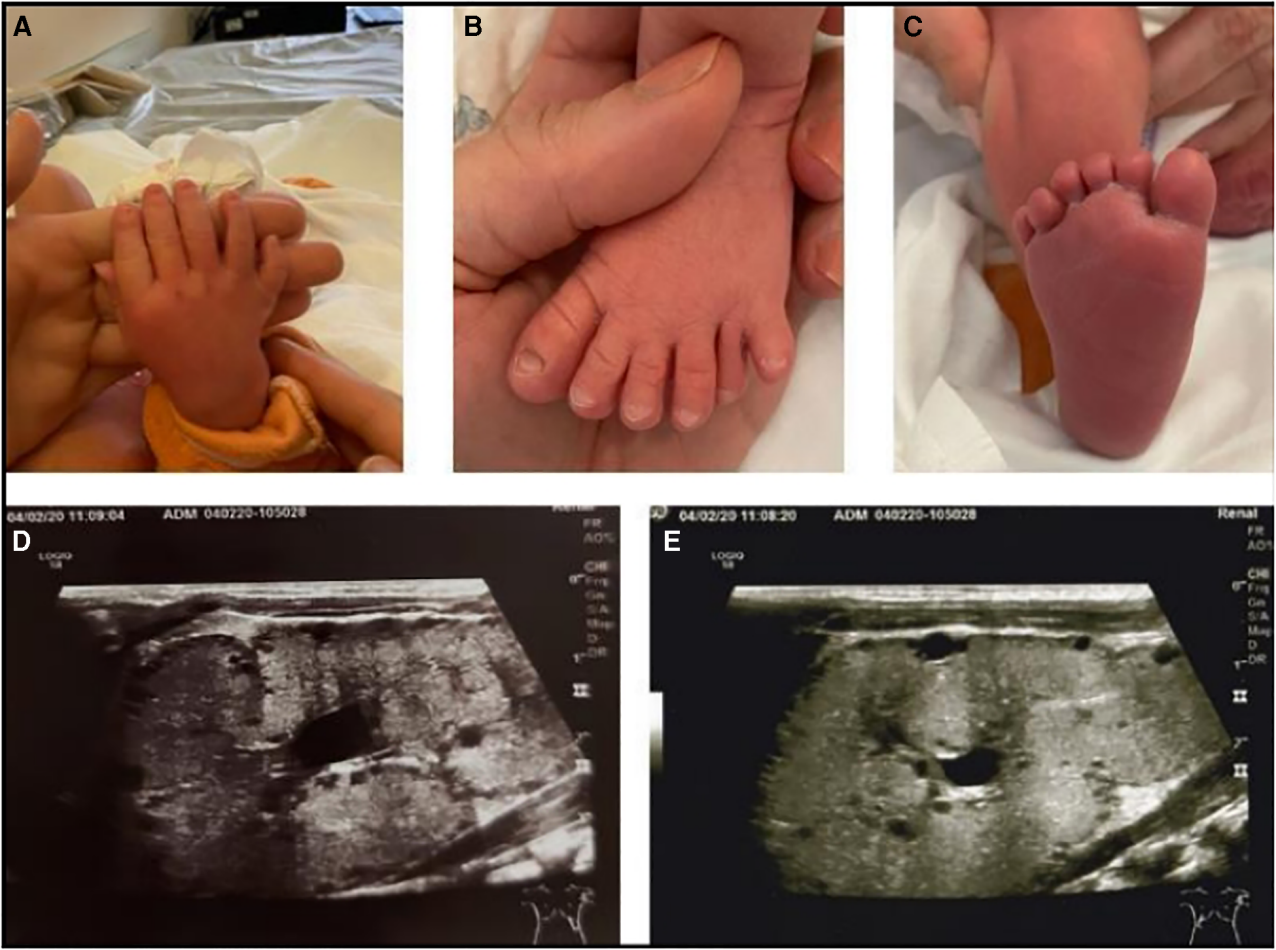
(**A**), hexadactyly of the right hand; (**B**), hexadactyly of the right foot; (**C**), hexadactyly of the left foot; (**D,E**), longitudinal ultrasound section through the left and right kidney.

Our patients include the proband, his brother and his sister. The proband is a male newborn, born in the 38th gestational week, with Apgar score 10/10, birth weight of 3,200 g and birth length of 49 cm. Fetal ultrasound made during pregnancy revealed renal pelvis dilatation, enlarged and hyperechoic kidneys with a large number of macrocysts, hexadactyly of the right hand and both feet, and syndactyly of 2nd and 3rd fingers. Based on these findings, autosomal recessive cystic kidney disease was suspected. His older brother, who was 18 months old at the moment of the proband's disease presentation, also had polycystic kidneys with the same type of hexadactyly and syndactyly (Figures 1A–C), while their older sister (5 years old) additionally presented with hydrometrocolpos and hydronephrosis at birth. We suspected that proband and his siblings have clinical phenotypes of overlapping symptoms of BBS and McKusick Kauffman syndrome. They were tested with a gene panel for 102 different ciliopathic genes. Two pathogenic missense variants, c.1277G > A (p.Cys426Tyr) and c.940A > G (p.Arg314Gly), were identified in BBS12 in each patient. These variants are on opposite chromosomes which supported the diagnosis of autosomal recessive BBS12 (BBS 12, MIM 615989). Both variants have been observed in patients with clinical features of Bardet-Biedl syndrome and classified as pathogenic (Variation ID: 347502 and 1337071).

All three affected members of the family were routinely followed during a two-and-a-half-year period in four-month intervals. Blood and urine tests, ultrasound of the urinary system, ultrasound of the abdomen, ultrasound of the heart and electroencephalogram were regularly monitored. Multidisciplinary supervision was conducted, including pediatric nephrologist, endocrinologist, cardiologist, neurologist as well as ophthalmologist, otorhinolaryngologist and clinical psychologist. The presenting symptoms of our proband were polyuria and polydipsia, with fluid intake over 2 L/day and urine osmolality under 110 mOsm/kg at the age of 1.5 years. Screening for proteinuria as well as measurements of arterial pressure were performed at each examination as leading indicators of CKD progression. Proband had proteinuria but the same wasn't detected in his brother's urine tests. Their sister had normal blood and urine tests. They were all normotensive during the whole follow-up period (Table 1.). Furthermore, all three patients had an almost identical ultrasound finding of the urinary system, described as diffusely hyperechoic parenchyma of both kidneys, without corticomedullary differentiation with numerous small cysts (Figures 1D, E). The sister additionally presented as a newborn with a malformation of the genitourinary system (vaginal atresia and hydrometrocolpos), but with the exception of polycystic kidney disease and the limb malformations described above, none of the patients developed other clinical manifestations of BBS during the mentioned follow-up period. However, routine monitoring of proband's renal function parameters showed a gradual decline with the development of third-grade chronic kidney disease (CKD) at the age of two years (Table 1). The same was observed in his brother's results with the development of second-grade CKD at the age of three. Their sister, on the other hand, does not express the same clinical presentation of renal disease and has a normal kidney function. Apart from the surgical correction of the described polydactyly, our proband is treated with an angiotensin-converting enzyme inhibitor (enalapril), sodium bicarbonate, calcium carbonate with vitamin D, and an iron supplementation as a part of the treatment of CKD. Up-to-date monitoring of possible CKD progression is foreseen and also a consideration of all available methods of replacement treatment if necessary. Although for now our patient, as well as his closest relatives, have not developed other possible clinical characteristics of BBS, further monitoring and follow-up are necessary.

**Table 1:** Renal function parameters during the follow-up period.

Age	BP	Creatinine	EGF	Proteinuria
(years)	(mmHg)	(µmol/L) (n. 15–37)	(Schwartz ml/min/1.72 m²)	(urine portion)
1.4	109/50	79	38	Alb < 3 mg/L (*n*. < 20)
				TP 33 mg/L
				UPCR 75 mg/mmol (*n*. < 50)
1.8	102/59	89	34	Alb 4 mg/L
				ACR 4.8 mg/mmol (*n*. < 3)
				TP 32 mg/L
				UPCR 38.5 mg/mmol (*n*. < 50)
2.1	103/69	85	37	Alb 4 mg/L
				ACR 5.4 mg/mmol
				TP 33 mg/L
				UPCR 44.6 mg/mmol (*n*. < 20)
				*β*-2 micro 4,820 ug/L
				(*n*. < 300)
2.4	104/65	91	37	Alb < 3 mg/L
				TP 26 mg/L
				UPCR 29.5 mg/mmol
				β-2 micro 4,820 ug/L
2.8	104/59	95	37	Alb 4 mg/L
				ACR 6.2 mg/mmol
				TP 32 mg/L
				UPCR 49 mg/mmol
				β-2 micro 4,960 ug/L
3.1	95/54	105	33	Alb 5 mg/L
				ACR 4.7 mg/mmol
				TP 39 mg/L
				UPCR 36.8 mg/mmol
				β-2 micro 4,800 ug/L
Brother				
3.2	103/61	47	79	Alb <3 mg/L
				TP 39 mg/dU (*n*. < 150)
				β-2 micro 348.8 ug/L (n. 33–363)
4.2	93/45	52	77	Alb < 3 mg/L (*n*. < 20)
				TP 53 mg/L
5	106/56	50	83	Alb 8 mg/dU (*n*. < 30)
				TP 43 mg/dU (*n*. < 150)
				β-2 micro 311.3 ug/L

BP, blood pressure; EGF, estimated glomerular filtration; Alb, albumin; TP, total protein; UPCR, urine protein to creatinine ratio; ACR, albumin to creatinine ratio; β-2 micro, β-2 microglobulin; *n*, normal referent range.

## Discussion

Bardet-Biedl syndrome belongs to the group of primary ciliopathies (6). It is a group of hereditary disorders, based on a genetic mutation characterized by impaired protein synthesis, which ultimately results in abnormal function and/or structure of primary cilia. Considering that primary cilia are microtubule-based organelles that extend from the apical surface of most mammalian cells, the possibility of multiorgan involvement is not surprising and therefore a great diversity in the clinical expression of the mentioned group of disorders (7). BBS is a „primary ciliopathy“, as opposed to secondary ciliopathies where the expression of the affected genes is not in the primary cilia and yet cysts are formed, probably due to cell proliferation and changing quality of environmental factors (6, 8).

Recent studies have identified at least 26 different genes whose mutation is associated with the development of BBS (*BBS 1-21*, *CEP164*, *SCAPER*, *SCLT1*) (3). Biallelic loss-of-function in previously mentioned genes is necessary in order for the disease to develop, which is the foundation of autosomal recessive inheritance. One of the eight genes encoding for the subunits of the octameric protein complex, BBSome (*BBS1, 2, 4, 5, 7, 8, 9, 18*) are most frequently affected by mutations in BBS patients. The second largest group of BBS patients have mutations in genes encoding chaperonins assisting the assembly of the BBSome (*BBS6/MKKS, 10, 12*) while the third most common group of patients has a mutation in *BBS3/ARL6*, a GTPase assisting the BBSome function (9–13).

In particular, all patients from our family are compound heterozygotes for the *BBS12* gene with two different mutations in each allel. Research suggests that genotypic diversity underlies the phenotypic variability of patients with BBS (14).

Bardet-Biedl syndrome is primarily characterized by retinal cone-rod dystrophy, obesity, postaxial polydactyly, cognitive impairment, hypogonadotropic hypogonadism and/or genitourinary malformations, and renal malformations and/or renal parenchymal disease. Due to the potential multiorgan involvement, systematic multidisciplinary follow-up of almost all pediatric subspecialists is required which is the reason why our patients perform routine nephrological, endocrinological, neurological, cardiological and ophthalmological diagnostics on a monthly basis (1).

Since it is known that it takes years for certain clinical characteristics of the syndrome to develop, the importance of a high degree of suspicion in oligosymptomatic patients (e.g., altered renal ultrasound with hexadactyly) and also the importance of genetic analysis of these patients in order to establish a timely diagnosis, regularly monitor and potentially prevent long-term complications, is not surprising (14, 15).

Even though a certain level of genotype-phenotype correlation in patients with BBS is well known, it should be emphasized that although all three of our patients are from the same family and have the same type of mutation detected (missense), they do not show a complete overlapping of the clinical picture. For instance, only proband's sister manifested with genitourinary malformations while all three patients had limb malformation and polycystic kidney disease. The similar was observed by Cherian et al. who have studied the clinical spectrum of 11 Saudi Arabian patients from four consanguineous families. In one of the families, in which five patients were affected, variability was observed not only in the overall clinical phenotype but also in the type of renal disease (16). Furthermore, after reviewing the literature, it was observed that a mutation in the *BBS12* gene can be presented with a different constellation of symptoms characteristic of BBS (17–19). According to the available literature, patients with this mutation can have higher cardiovascular risks than those with mutation in *BBS1* gene, since patients with mutations in BBS1 gene show low frequency of renal anomalies (potential cause of arterial hypertension) and generally present with milder phenotype while patients with mutations in *BBS12* often present with obesity (associated with hyperlipidemia and glucose intolerance) (3, 5).

Recent metanalyses suggest that the identity of the causative gene and the type of the mutation can partially predict the clinical outcome of the disease. But to what extent? It is known that mutations in *BBS1*, *BBS2*, and *BBS10* gene are the most common ones among BBS population (making around 50% of all cases) and what is also shown is that patients with mutations in *BBS2* or *BBS10* gene have higher penetrance of renal anomalies and limb malformations (polydactyly) than patients with mutations in *BBS1* (3). Given that renal anomalies are one of the main characteristics of BBS and that potential renal failure is a life-threatening complication, the clinical significance of the exact determination of the affected gene and the type of mutation is clear. The question arises whether the mentioned genotypic definition can also predict the outcome of renal impairment, or rather, the probability of developing CKD (20, 21)?

The renal phenotype of BBS varies from urinary concentration defects to structural anomalies such as horseshoe, ectopic, duplex, or absent kidneys; or polycystic kidney disease, hydronephrosis and vesicoureteral reflux (2, 5). Although all three of our patients have the same structural type of kidney disease, at the same time they do not express the same degree of renal function impairment. For example, our proband has developed 3rd grade CKD at the age of two years, his brother has developed 2nd grade CKD at the age of four, while their sister who is at the age of 5 years has a completely normal kidney function. Some of the previous studies managed to define genes that are associated with a higher risk of progression to CKD (22). A study by Forsyth and his associates identified an increased prevalence of severe kidney disease defined as an estimated glomerular filtration rate (eGFR) <45 ml/min/1.73 m^2^ in individuals with *BBS2*, *BBS10*, and *BBS12* compared to individuals with *BBS1*. Also, in the same study, homozygous and compound heterozygous individuals with truncating variants were more likely to be associated with severe kidney disease than those with two missense variants implicating that neither the type of mutation is prognostically negligible. The similar was observed in some recent studies indicating the importance of timely and up-to-date monitoring of renal function in all patients with BBS (20–23).

What is interesting to point out is that the meta-analyses that studied the genotype-phenotype correlation of other symptoms of BBS did not find such a strong association as is the case with renal anomalies. This is possibly attributed to the importance of the preservation of the core of the BBSome for the structure of the kidney, considering that the mutations in *BBS2*, *BBS7*, or *BBS9* induced a very high incidence of renal anomalies and yet is known that those genes encode proteins fundamental for establishing the core of the BBSome (3).

The question arises as to what factors influence the diversity of clinical expression in patients with BBS, especially in those who have the same genetic defect caused by the same type of mutation. What underlies intrafamilial phenotypic variability as has been observed in our case report? Penetrance variability, epistasis phenomenon as well as environmental factors need to be taken into consideration (22, 24)? The mechanisms underlying clinical heterogeneity are complex and can possibly be explained by multiple loci, ubiquitous gene expression as well as multiple interaction of proteins encoded by genes involved in BBS (25). Considering that the exitance of the genes necessary for the normal function and structure the primary cilia is known and yet they are not causally related to BBS, perhaps their research would provide a clearer insight into the many of the possible disease phenotypes. The clinical application of these molecular processes remains to be elucidated (6, 7).

## Conclusion

We report a very rare case of a family with three members with Bardet-Biedl syndrome. All three patients have the same type of mutation, they are all compound heterozygotes for *BBS12* gene. However, they express a mild variety in clinical symptoms but at the same time a great variety in kidney function. We believe that the presentation of this case will further contribute to the understanding of the connection between genotype and phenotype in patients with this rare syndrome, especially those with rare genetic mutations within the same family. The importance of genetic analysis lies within the possibility of establishing timely diagnosis, even in oligosymptomatic patients who have not yet fully developed the whole spectrum of clinical expression, which sometimes takes years (obesity, hypogonadism, intellectual disabilities, etc.). Considering that CKD is one of the main determinants of morbidity and mortality in these patients and that it's onset is insidious and possible within the first year of life, we emphasize the importance of a high degree of suspicion and up-to-date follow-up of all patients with abnormal ultrasound findings, especially those with hyperechoic and polycystic kidneys (20, 22, 23). However, a diagnostic test that would predict the degree of kidney impairment even after a clearly established genotype does not exist for now, and further research in this area will be necessary. Long-term follow-up of these patients will provide insight into the progression of kidney disease as well as the development of other features of BBS over time, and can serve as a basis for subsequent retrospective and prospective research.

## Data Availability

The datasets presented in this study can be found in online repositories. The names of the repository/repositories and accession number(s) can be found in the article.
